# Biologic Tuberoplasty With an Acellular Dermal Allograft for Massive Rotator Cuff Tears

**DOI:** 10.1016/j.eats.2021.03.016

**Published:** 2021-06-20

**Authors:** Raffy Mirzayan, Gabriel Bouz

**Affiliations:** a Department of Orthopaedics, Kaiser Permanente Southern California, Baldwin Park; b Department of Orthopaedics, University of Southern California Keck School of Medicine, Los Angeles, California, U.S.A.

## Abstract

We present the technique of biologic tuberoplasty, where an acellular dermal allograft is used to cover the tuberosity in patients with massive irreparable cuff tears to prevent bone-to-bone contact between the tuberosity and acromion when active elevation is attempted. This technique can be performed in patients with massive rotator cuff tears who are low-demand and have significant medical comorbidities, poor bone quality, or who are not candidates for a reverse shoulder arthroplasty or who cannot tolerate a lengthy rehabilitation protocol. It is less time-consuming than a superior capsule reconstruction and more bone-sparing than traditional tuberoplasty. Patients with massive rotator cuff tears involving the supraspinatus and a portion of the infraspinatus lose their force couple, leading to superior humeral head migration with abutment of the tuberosity against the acromion upon deltoid activation. The center of rotation moves superiorly with deltoid contraction, developing an acromiohumeral articulation. This results in bone-to-bone contact between the acromion and humerus, leading to pain and acetabularization of the acromion over time. Coverage of the tuberosity with the acellular dermal allograft acts as an interpositional tissue and prevents bone-to-bone contact between the tuberosity and acromion.

Patients with massive rotator cuff tears involving the supraspinatus and a portion of the infraspinatus lose their force couple, leading to superior humeral head migration with abutment of the tuberosity against the acromion upon deltoid activation.^1^ The center of rotation moves superiorly, with deltoid contraction developing an acromiohumeral articulation.^1^ This results in bone-to-bone contact between the acromion and humerus, leading to pain and acetabularization of the acromion over time.^2^ Several procedures have been reported for the treatment of massive irreparable rotator cuff tears, including arthroscopic debridement, biceps tenodesis or tenotomy, partial repair, tendon transfers, superior capsular reconstruction (SCR), balloon arthroplasty, and reverse total shoulder arthroplasty.^3^ The recently described SCR is an extensive operation with a long postoperative rehabilitation program indicated for patients with symptomatic massive rotator cuff tears with poor function who are too young for a reverse total shoulder arthroplasty.^4^ Biomechanical studies have shown that the dermal allograft used for SCR acts as a static stabilizer of the humeral head, keeping the humeral head centered on the glenoid and therefore restoring the normal biomechanics of the glenohumeral joint.^5^

For patients with significant comorbidities who are unable to undergo an extensive operation, the options are limited. Many of these patients have poor bone quality and are at high risk for rotator cuff retear, graft tear, or anchor pull-out.^6^ Mirzayan et al.^7^ recently noted that in patients who had undergone SCR but on postoperative magnetic resonance imaging had graft tear leaving the greater tuberosity covered had equivalent visual analog scale scores and functional outcomes as patients with intact grafts. They termed this observation as the “biologic tuberoplasty effect” because the authors felt the dermal allograft healed to the tuberosity was preventing bone-to-bone contact between the acromion and tuberosity.

This article describes the technique of biologic tuberoplasty using an acellular dermal allograft, providing a quicker, bone-sparing, less time-consuming option for patients with massive rotator cuff tears who are low-demand and have significant medical comorbidities (Table 1).

**Table 1:** Advantages and Disadvantages

Advantages
1. Lower cost relative to prosthetic replacement.
2. Decreased time under anesthesia for high-risk patients.
3. Addition of acellular dermal allograft adds further cushion and preservation of bone stock compared to tuberoplasty alone.
4. Significant pain relief.
5. Avoidance of complications of prosthetic replacement.
6. Low-demand postoperative rehabilitation.
7. Does not increase difficulty of prosthetic replacement at later time (does not “burn bridges”).
Disadvantages/risks and/or limitations
1. Cost of dermal allograft.
2. Use of implant, technical risk of foreign body reaction.
3. Disadvantage compared with superior capsule reconstruction is that it does not restore normal glenohumeral joint kinematics—it is intended solely as a pain reliever.

## Surgical Technique (With Video Illustration)

The tips and pearls of this technique are listed in Table 2, and the technique is demonstrated in Video 1. The patient is positioned in lateral decubitus, ensuring all prominences are well-padded and secured. The extremity is adequately prepped and draped in sterile fashion. The arm is held in a shoulder distraction system with 10 to 20 pounds of traction for optimal visualization of the glenohumeral joint. A posterior portal is created and a diagnostic arthroscopy is performed. Additional portals, including a lateral “working” portal, a posterolateral “viewing” portal, and an anterior portal, are created. Attention is then turned to the tuberosity. A combination of arthroscopic shaver and electrocautery is used to debride any soft tissue remaining on the footprint. A high-speed burr in reverse mode is used to remove any osteophytes and any bony prominences until underlying bleeding osseous bone is visualized. Care is taken to spare as much bone as possible and to avoid aggressive resection of the tuberosity.

**Table 2:** Pearls and Pitfalls

Pearls
1. Use a high-speed burr in reverse mode when abrading the tuberosity to minimize bone resection.
2. Use a measuring device to measure the length and width of the tuberosity.
3. Use a large cannula without a dam to pass graft. If not available, use a 10-cc syringe with the tip cut off.
4. Pass mattress sutures with the repair stitch from each anchor in the edge of the graft.
5. Sequentially tension the pulling stitches to reduce graft into subacromial space.
6. Once graft is secured to medial edge, pull each limb of FiberTape individually to reduce entanglement of the FiberTapes and improved visualization.
Pitfalls
1. Do not use suture passing device to pass sutures through graft. Use a reverse cutting needle instead.
2. Don't undersize the graft when cutting it out of the larger piece of graft.

**Fig 1:**
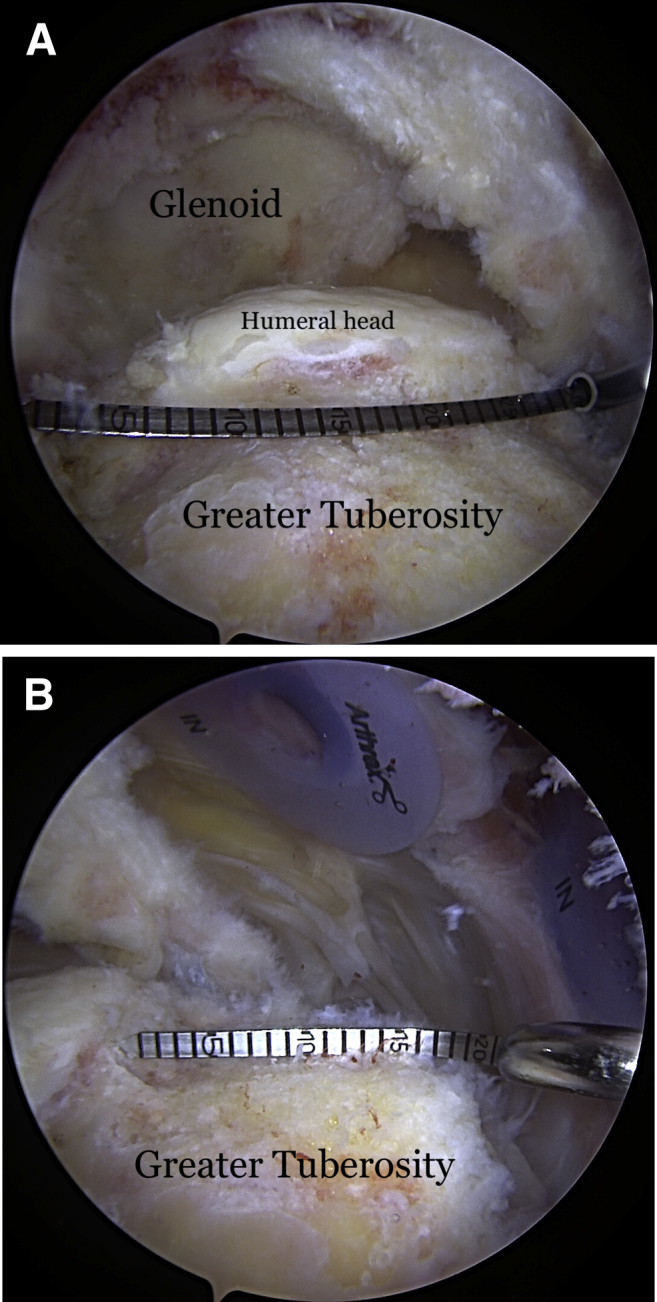
(A) View from lateral portal of a right shoulder with the patient in lateral decubitus position demonstrating the measurement of the length of the tuberosity. (B) View from posterior portal of a right shoulder demonstrating the measurement of the width of the tuberosity.

**Fig 2:**
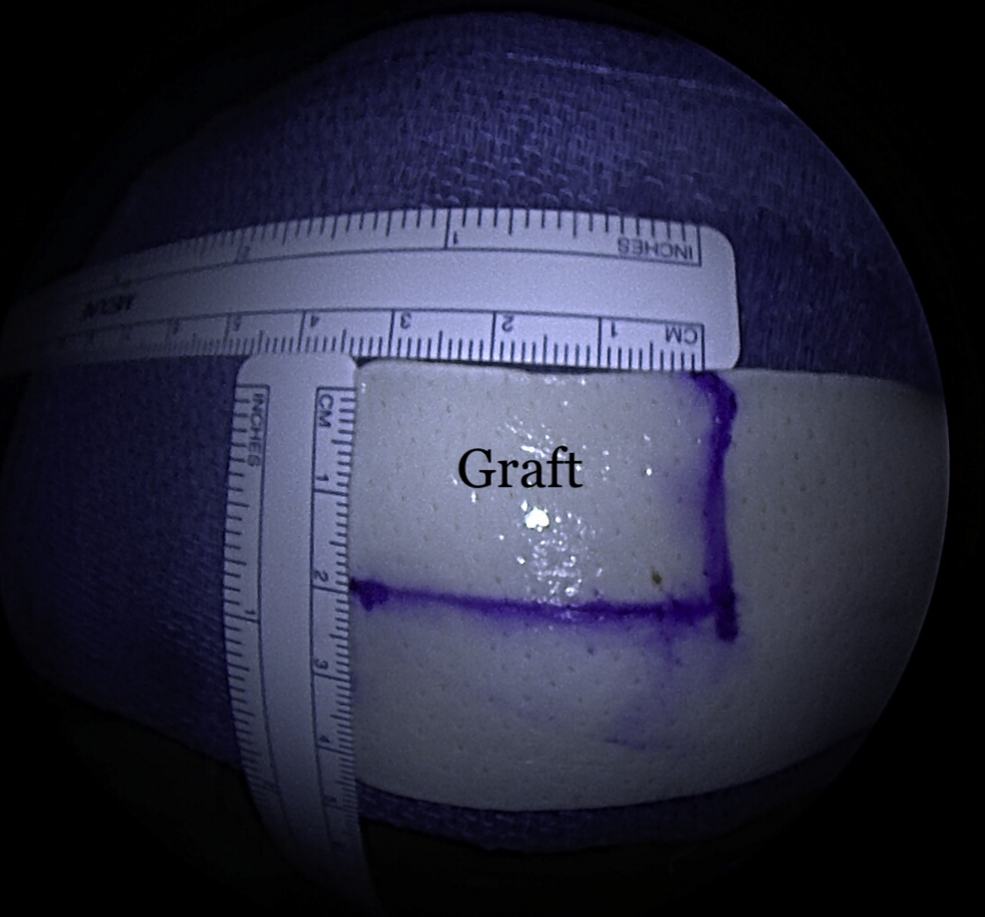
A 30-mm × 20-mm piece of the graft is obtained from a 3-mm thick ArthroFlex 301 (LifeNet Health, Virginia Beach, VA) dermal allograft.

**Fig 3:**
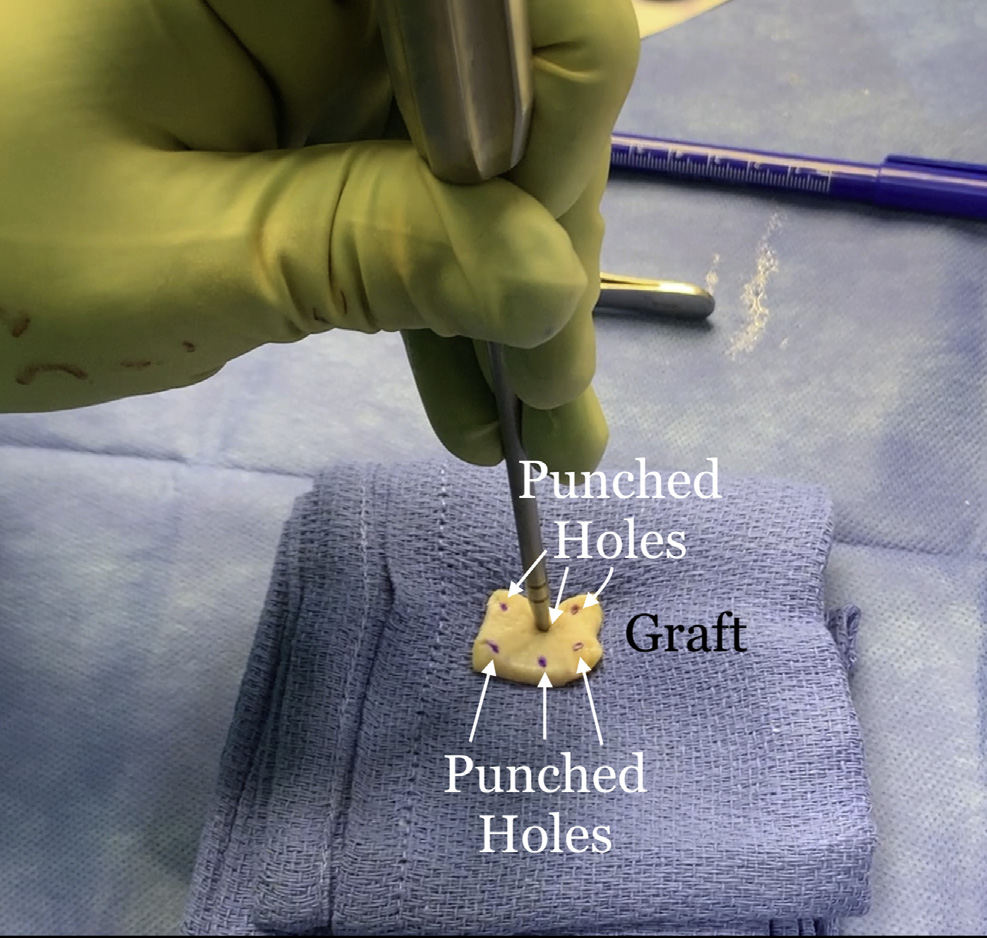
Holes are punched in the graft to allow for easier suture/FiberTape passage.

**Fig 4:**
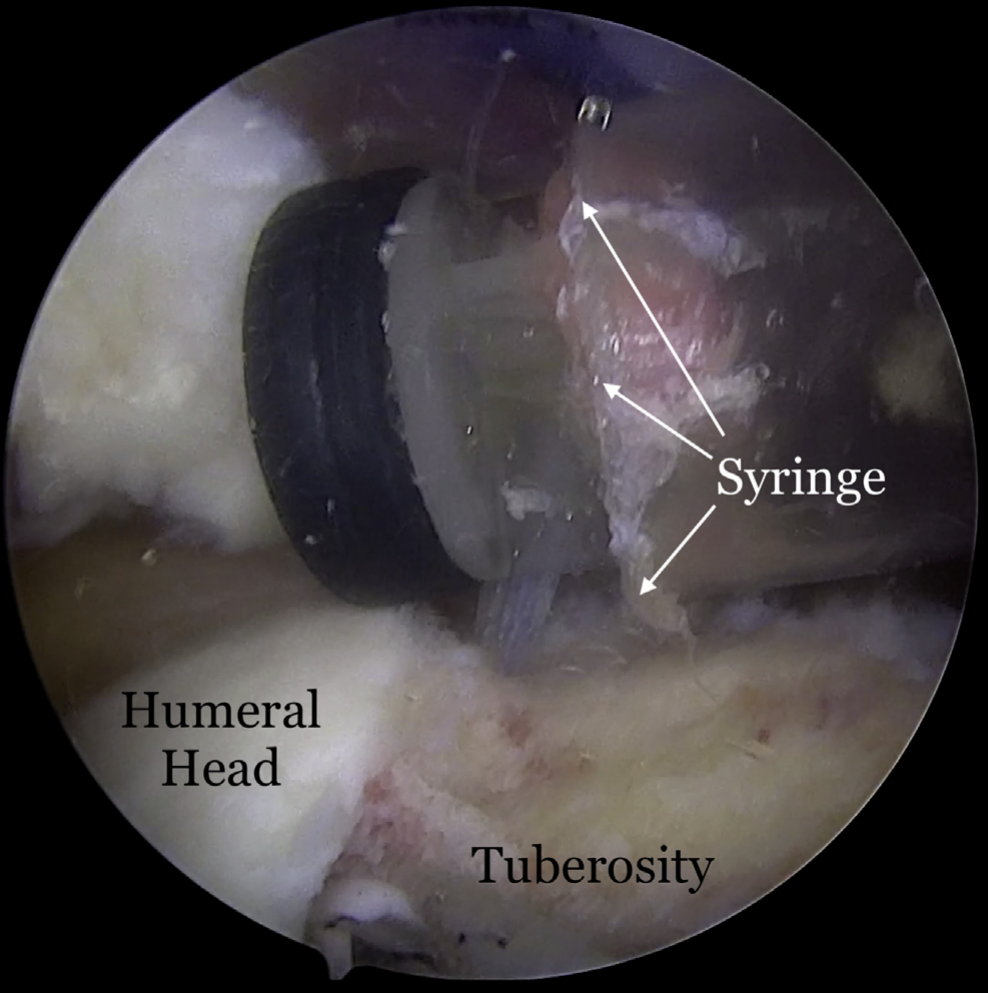
View from posterior portal of a right shoulder with the patient in lateral decubitus position demonstrating the insertion of a 10-cc syringe with its tip cut off (white arrows).

Once the graft site has been prepared, measurements of the graft dimensions (length and width) are achieved with the use of an arthroscopic measurement probe (Fig 1). On the back table, a 3-mm thick acellular dermal allograft (ArthroFlex 301; LifeNet Health, Virginia Beach, VA) is measured and cut under tension according to the measured length and width to ensure adequate coverage of the tuberosity (Fig 2). Depending on the size of the desired graft, 2 or 3 puncture holes are made at the medial and lateral aspects of the graft for easier passage of sutures and FiberTape (Arthrex, Naples, FL) (Fig 3). Three 4.75-mm knotless SwiveLock anchors (Arthrex) are placed in the anteromedial, central, and posteromedial aspects of the tuberosity footprint just lateral to the articular margin of the humeral head. Given the challenge of shuttling large grafts into the subacromial space through most commercially available cannulas which contain a dam, the senior author uses a 10-cc syringe with its end cut off. The tip of the 10-cc syringe is cut and introduced into the anterolateral portal for easier passage of the graft (Fig 4).

**Fig 5:**
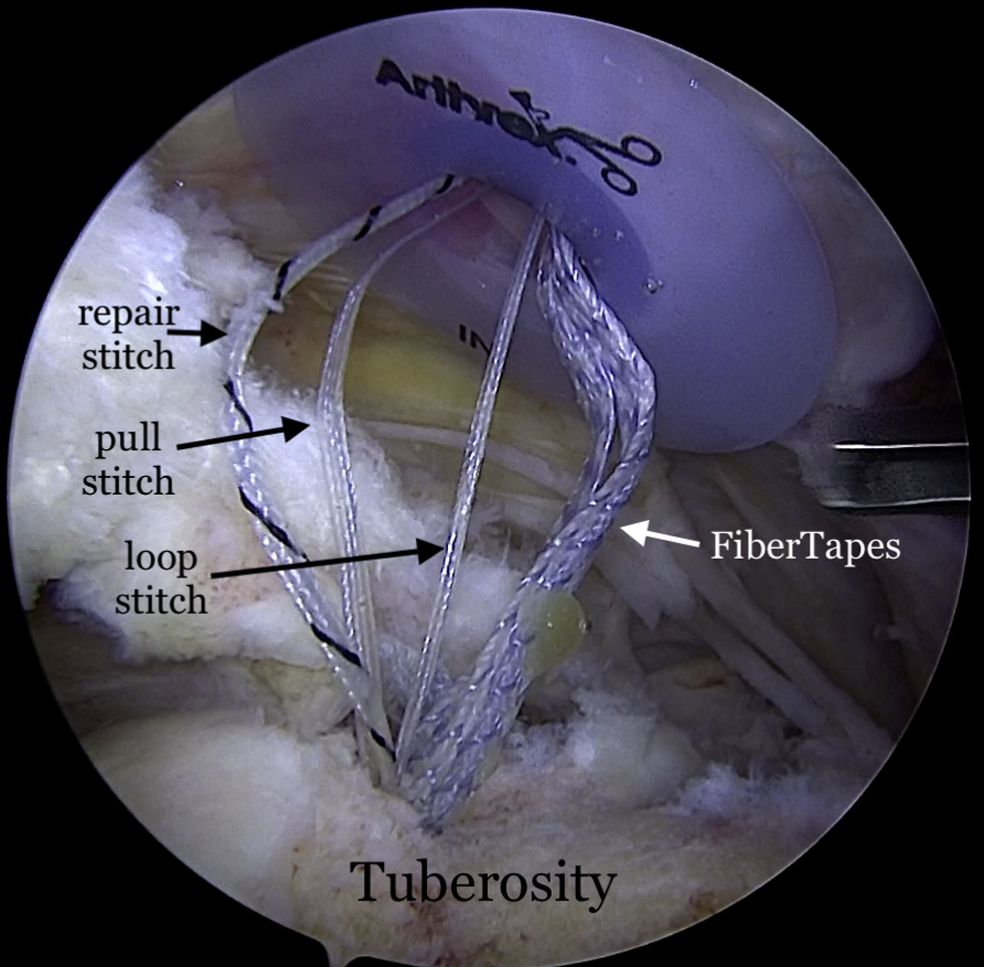
View from posterior portal of a right shoulder with the patient in lateral decubitus position. The 2 FiberTapes, the repair stitch, and the loop end of the shuttle stitch from each anchor are taken out of the syringe, leaving behind the pull end of the shuttle stitch.

**Fig 6:**
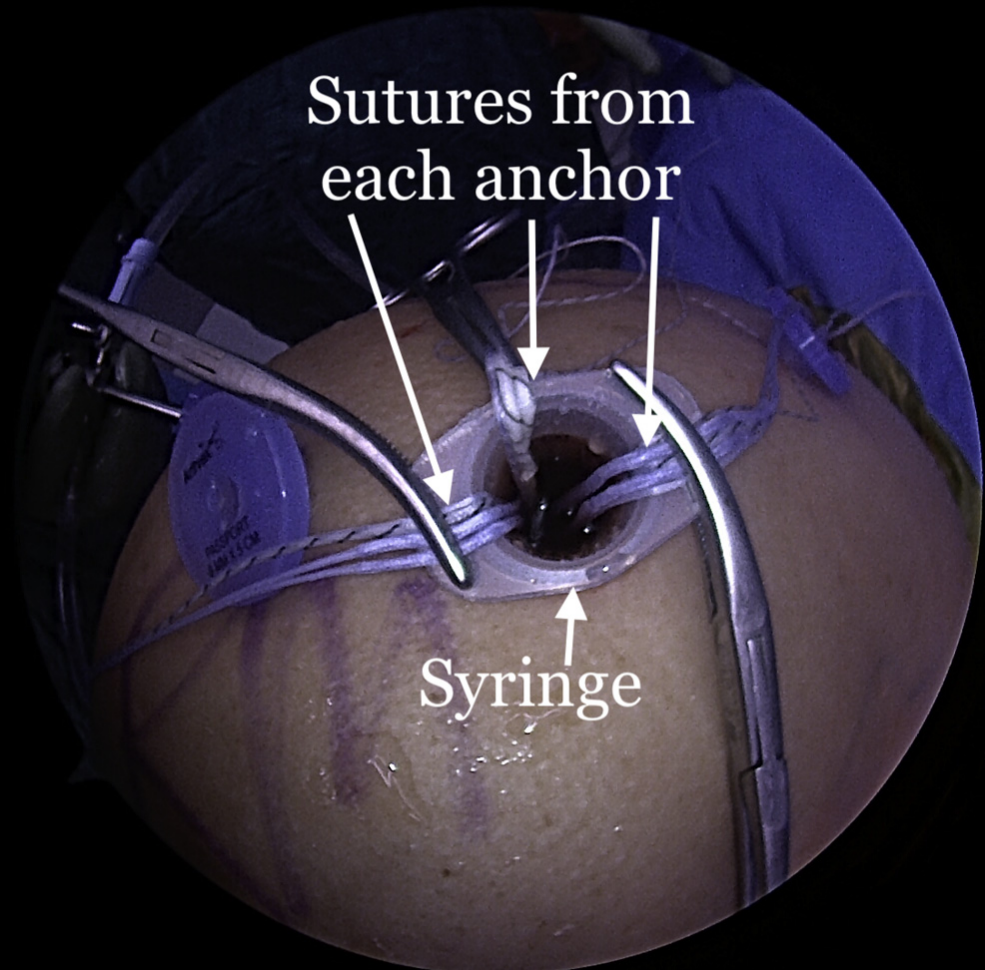
Exterior view of a right shoulder with the patient in the lateral decubitus position. The sutures from each anchor are kept separate and clamped to the syringe for suture management.

**Fig 7:**
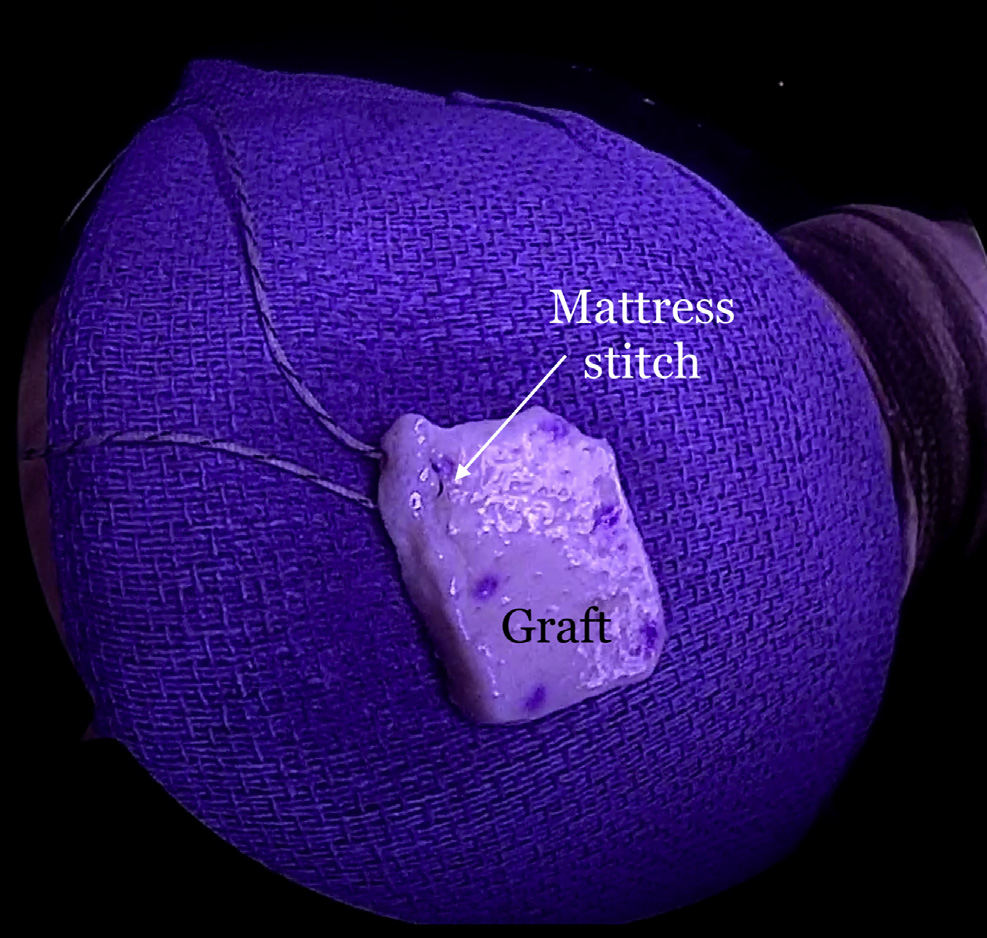
Exterior view of a right shoulder with the patient in the lateral decubitus position. The repair stitch is passed in a mattress fashion at the edge of the graft. A towel is placed on the patient's arm near the syringe cannula to pass the sutures and FiberTapes through the graft.

**Fig 8:**
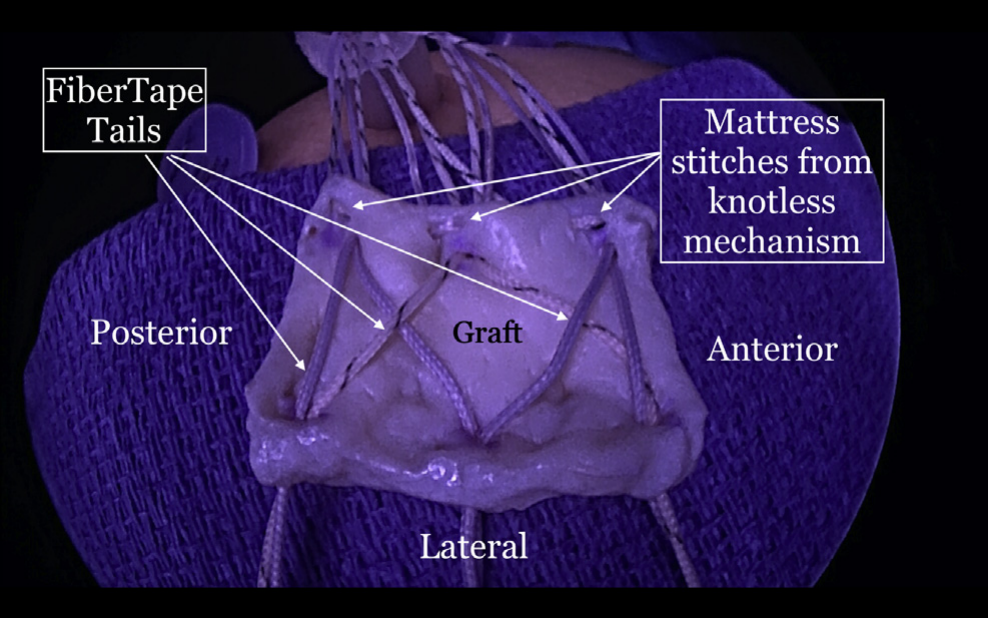
Exterior view of a right shoulder with the patient in the lateral decubitus position. The final suture passage and graft preparation before insertion into the subacromial space are shown here. The mattress sutures at the medial edge are passed with the repair stitch from each anchor. The FiberTapes are passed through the pre-punched holes on the medial edge of the graft and then passed back down through the graft in an expanded speedbridge configuration. This allows better control of the lateral portion of the graft when the lateral row anchors are being inserted.

**Fig 9:**
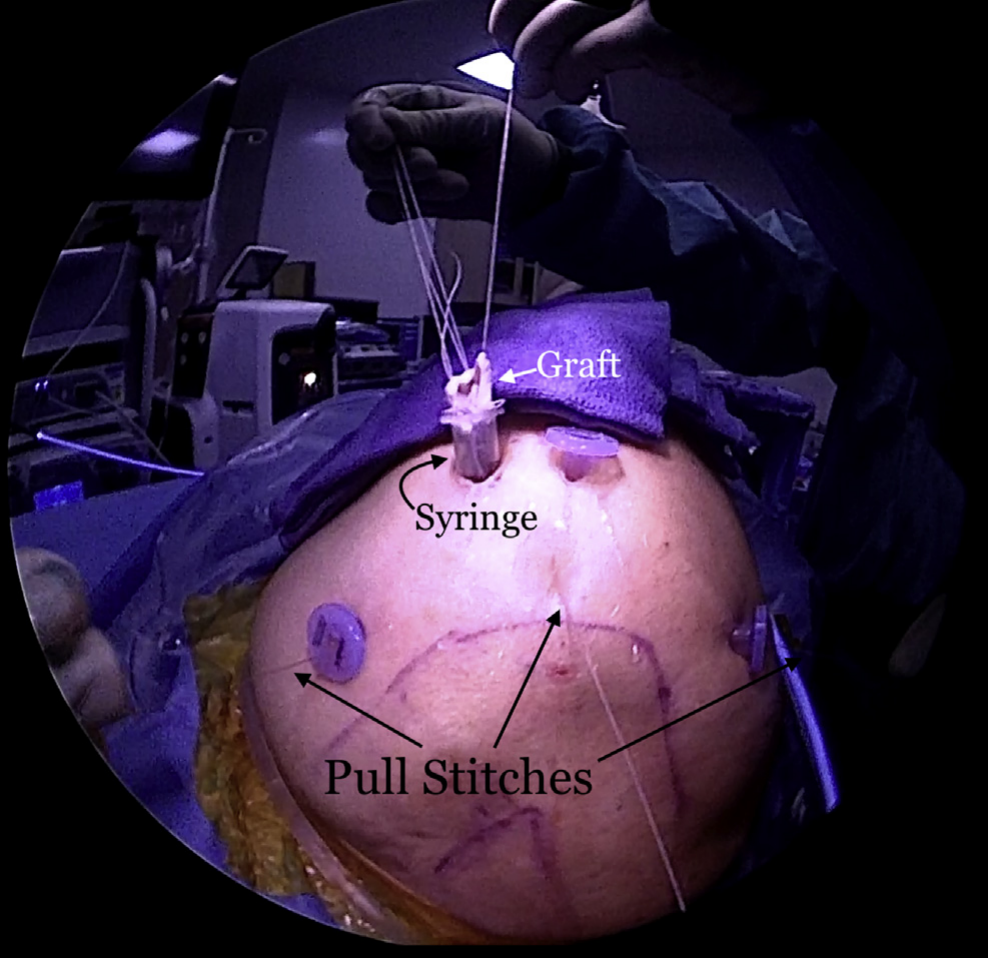
Exterior view of a right shoulder with the patient in the lateral decubitus position. The pull stitches are pulled sequentially, pulling the graft into the subacromial space through the syringe.

**Fig 10:**
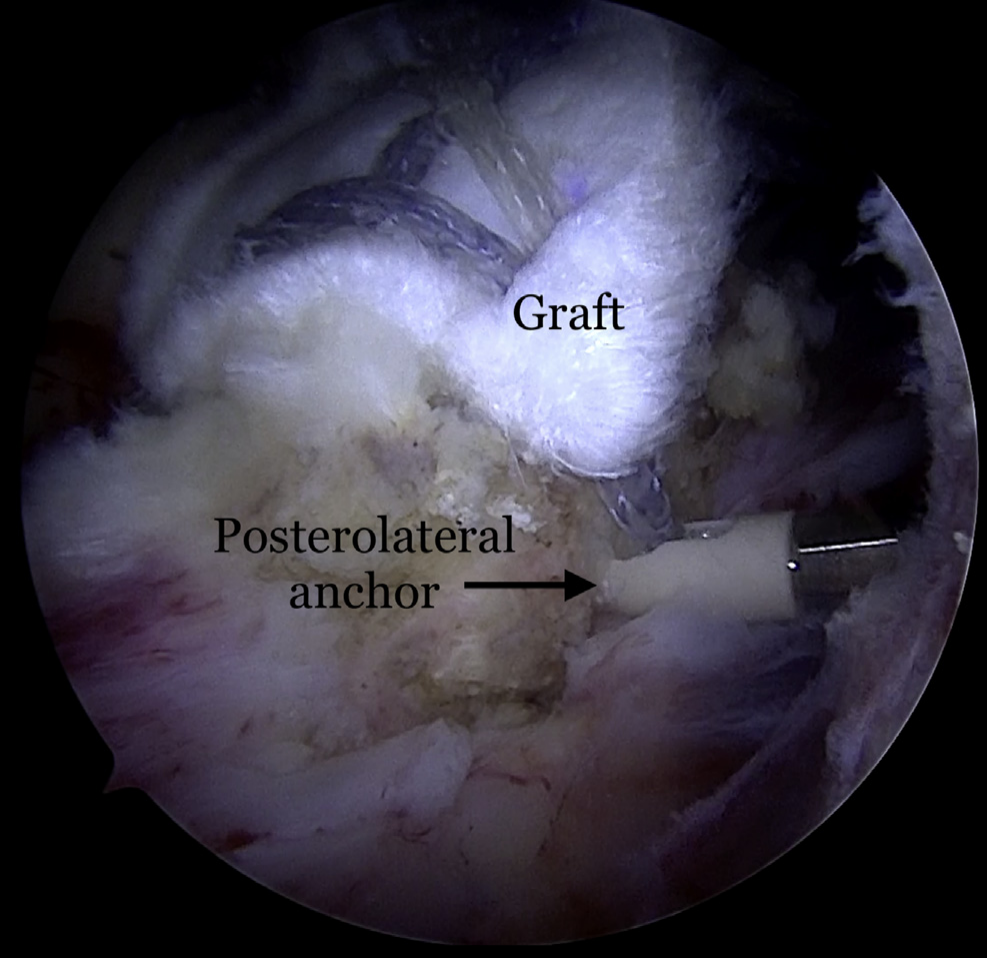
Right shoulder viewed from the posterior portal with the patient in the lateral decubitus position demonstrating the insertion of the posterolateral anchor placement.

**Fig 11:**
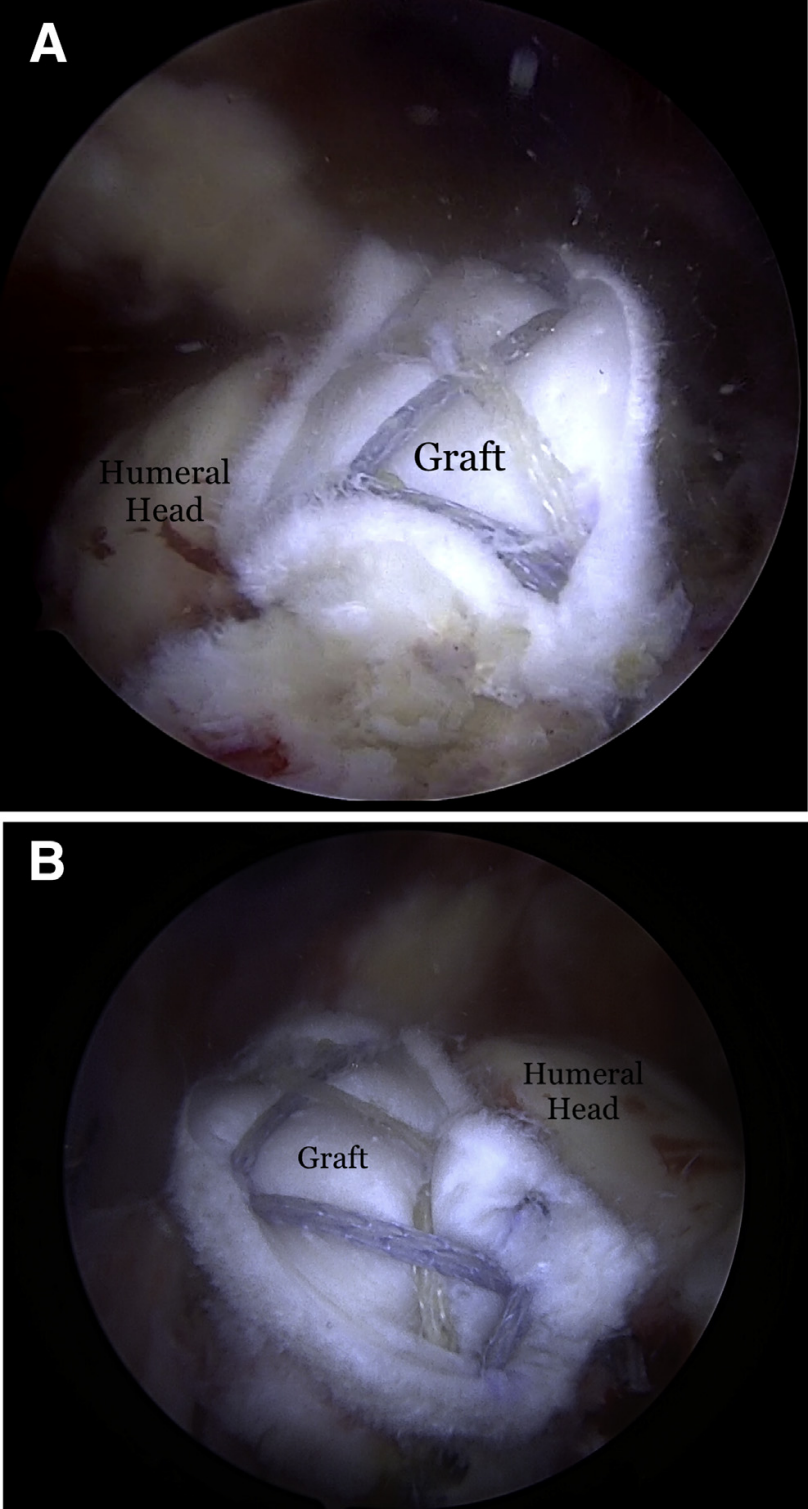
(A) Final graft placement and fixation seen in the right shoulder viewed from the posterior portal with the patient in the lateral decubitus position and (B) viewed from the anterior portal.

The repair stitch and the looped end of the shuttle stitch from each anchor are then retrieved through the syringe, leaving behind the pull end of the shuttle stitch (Fig 5). The sutures from each anchor are grouped and separately clamped to the edges of the syringe (Fig 6). The repair stitch from each anchor is passed in a mattress fashion from the edge of the graft using a free needle (Fig 7). The repair stitch is then passed through the looped end of the shuttle stitch and folded over at the designated blue-colored marker on the stitch. The pull stitch is then shuttled bringing the repair stitch into the knotless mechanism and locking it in place. This step is repeated for each anchor. The FiberTape sutures (Arthrex) from each anchor are passed through the pre-punched holes. One of the FiberTape sutures from the anterior-most and posterior-most holes on the medial edge of the graft are passed through the central pre-punched hole on lateral edge of the graft. One of the 2 central FiberTape sutures is passed over to the anterior hole on the lateral edge of the graft. The remaining central FiberTape suture is passed over to the posterior hole on the lateral edge of the graft, creating an expanded speed bridge pattern (Fig 8). This allows for better control of the graft when the lateral anchors are placed for fixation. The pull end of the shuttle stitches in each of the anchors are then sequentially pulled, shortening the mattress passes, thus pulling the graft into the shoulder to the medial edge of the footprint (Fig 9). Once satisfied with the placement of the medial aspect of the graft, the FiberTape sutures are retrieved from posterolateral, central and anterolateral aspects of the graft. Each pair of FiberTape sutures is then loaded into a 4.75-mm SwiveLock anchor to fixate the graft laterally (Fig 10). Better control of the graft is achieved having passed the FiberTape sutures through the lateral edges of the graft. Final visualization of the graft from posterior, lateral, and anterior portals is performed to ensure no dogears and secure placement of the graft over the tuberosity preventing bone-on-bone contact between the humeral head and the acromion (Fig 11). The patient is placed in an abduction sling for comfort until the first postoperative visit. The sling is removed, and pendulum exercises are initiated. Patient can be weight-bearing as tolerated with initiation of passive range-of-motion beginning after the third week. Active range-of-motion and strengthening exercises are recommended to start 6 weeks after the procedure. The patient is expected to have full, pain-free range of motion by 3 months.

## Discussion

The described technique of biologic tuberoplasty provides a viable option for patients with massive irreparable rotator cuff tears who cannot tolerate a long, extensive operation or lengthy rehabilitation, who may have poor bone quality, or who may not be a candidate for shoulder arthroplasty. This procedure has several advantages especially for elderly, low-demand patients with significant medical comorbidities but with functional mobility, whose primary goal is pain relief (Table 1).

Patients with massive rotator cuff tears involving the supraspinatus and a portion of the infraspinatus lose their force couple leading to superior humeral head migration with abutment of the tuberosity against the acromion upon deltoid activation.^1^ Burkhart^1^ studied 12 shoulders with massive, irreparable rotator cuff tears fluoroscopically. He recognized 3 kinematic patterns: stable, unstable, and captured fulcrum. Shoulders that had the force couple maintained had a stable fulcrum with the humeral head centered on the glenoid. Shoulders with an unstable fulcrum had a tear large enough where the force couples were disrupted and allowed for anterior and superior translation of the humeral head with attempted active elevation which resulted in little more than a shrug. Shoulders with a captured fulcrum developed an acromiohumeral fulcrum with deltoid activation because the center of rotation moves superiorly with deltoid contraction developing an acromiohumeral articulation.^1^ This results in bone-to-bone contact between the acromion and humerus, leading to pain and acetabularization of the acromion over time.^2^

Fenlin et al.^8^ first described the technique of tuberoplasty, which involved an aggressive resection and rounding of the tuberosity resulting in a new “acromiohumeral articulation.” The authors reported improvement of functional outcomes and significant pain relief at a mean of 27 months' follow-up. Several studies have demonstrated short-to mid-term benefits of tuberoplasty, including pain relief, improvement in functional outcomes, and at times, improvement in shoulder range-of-motion.^9^^,^^10^ In patients with symptomatic massive rotator cuff tears who underwent arthroscopic tuberoplasty and concomitant acromioplasty, Lee et al.^9^ noted significant improvement in functional scores with 81% of patients reporting good or excellent outcomes, not being affected by age, sex, and preoperative mobility. In a more recent study with mean follow-up of 8 years, Park et al.^11^ noted significant pain relief and improvement in UCLA and Constant scores despite interval decrease in acromiohumeral distance over time. The theory behind the pain relief benefit of the tuberoplasty is associated with decreasing the distance between the acromion and humeral head, relieving any impingement that may be occurring in patients with deficient rotator cuffs. The traditional tuberoplasty is aggressive and entails significant bone removal or practically resection of the tuberosity, making it more rounded to allow it to slide under the acromion without significant abutment.

The interest in combining the placement of an acellular dermal matrix with a minimal tuberoplasty stemmed from the findings of Mirzayan et al.,^7^ which demonstrated no difference in visual analog scale and American Shoulder and Elbow Surgeons Shoulder Score in patients after SCR with intact grafts compared with those who sustained a graft tear with the graft leaving the tuberosity covered. The remaining graft over the tuberosity served as an interpositional cushion between the acromion and the humeral head, further decreasing contact pressures at the acromiohumeral articulation. The authors had performed a gentle abrasion of the footprint without resecting a significant portion of the tuberosity. Ravenscroft et al..^12^ recently described a technique of bursal acromial reconstruction where a dermal allograft is secured to the undersurface of the acromion with sutures and also acts as an interpositional tissue preventing bone to bone contact between tuberosity and acromion.

**Fig 12:**
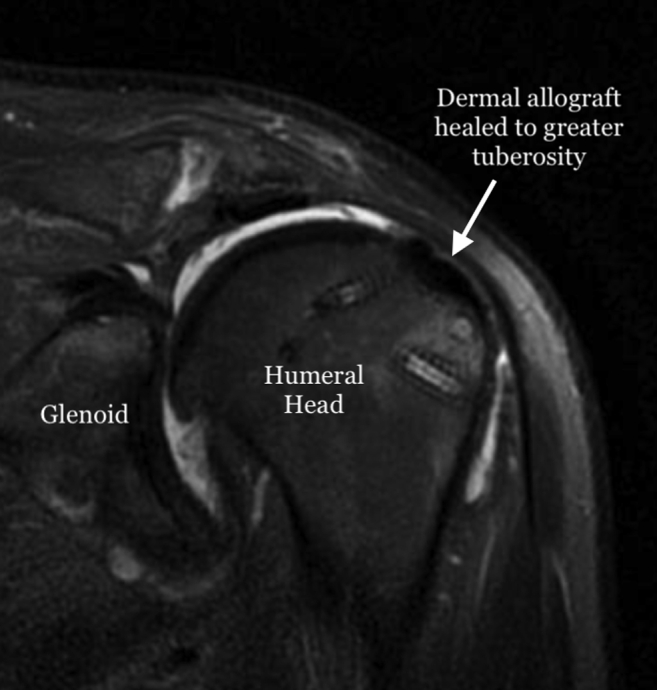
T2-weighted fat-saturated magnetic resonance imaging scan of a left shoulder 3 months after surgery demonstrating healing and incorporation of the graft to the greater tuberosity.

The use of autologous graft or acellular dermal allograft as spacers in patients with degenerative joint disease has been explored in various orthopaedic procedures in the hand, wrist, elbow and ankle.^13^, ^14^, ^15^, ^16^, ^17^, ^18^ Advantages of using acellular dermal allograft include no donor site morbidity, preservation of bone stock for an eventual prosthetic replacement, pain relief and improved range-of-motion.^13^^,^^14^^,^^17^^,^^18^ In patients with massive rotator cuff tears, who are too young or are poor candidates for prosthetic replacement, the described "biologic tuberoplasty" procedure provides the benefits of pain relief, maintained range-of-motion, and avoidance of a high-risk operation and lengthy rehabilitation (Table 3). A postoperative MRI 3 months following this procedure demonstrates a healed and incorporated graft (Fig 12).

**Table 3:** Indications and Contraindications

Indications
1. Massive, irreparable rotator cuff tear.
2. Poor bone quality which can lead to anchor pull out from the glenoid when attempting a superior capsule reconstruction.
3. Older patient (>70 or 75 years of age) who is not a candidate for arthroplasty.
4. Avoid lengthy rehabilitation.
5. Avoid lengthy surgery.
Contraindications
1. Active or previous infection.
2. Grade III-IV chondromalacia of the glenohumeral joint.

## Supplementary data

Video 1 The procedure is demonstrated on a right shoulder with the patient in the lateral decubitus position. A burr is used to remove any osteophytes or hypertrophy of the tuberosity until a bleeding cancellous surface is achieved. The length of the tuberosity is measured at 30 mm and the width at 20 mm. An ArthroFlex that is 3 mm thick is then measured to these dimensions and cut to size. Three puncture holes are made and also punched to widen them in the medial and the lateral edges of the graft for easier passage of FiberTape sutures. Knotless SwiveLocks are then placed in the anteromedial, posteromedial, and central aspect of the tuberosity footprint. A 10-cc syringe with the end cut off is used to allow for easier passage of the graft. The FiberTapes, repair stitch, and loop stitch are then retrieved through the syringe from each anchor, leaving behind the pull stitch from each anchor. The sutures from each anchor are kept separated and clamped to the syringe for suture management. The repair stitch from each anchor is then passed in a mattress fashion from the edge of the graft and is then passed through the loop of the loop stitch and folded over. The pull stitch from that anchor is then pulled, bringing the repair stitch into the knotless mechanism and locking it in like a finger trap so it cannot back out. This is performed for each anchor. The mattress stitches will be used to pull the graft into the shoulder. The FiberTapes from each anchor are then passed through the pre-punched holes. The FiberTapes are then passed back through the lateral edge of the graft in an expanded speedbridge configuration. This will allow better control of the graft when lateral row anchors are placed for fixation and will eliminate dog ears in the graft. The pull sutures from each anchor are then sequentially pulled, allowing the mattress passes to shorten and thus reducing the graft into the shoulder. The graft can be seen entering the subacromial space. The pull stitches are pulled until the graft is secured to the medial edge of the footprint. Although there appears to be disorganization of sutures, when the FiberTape limbs are pulled individually, the slack is taken out of them and the graft and sutures become more manageable. The posteromedial corner of the graft is grasped and reduced to that corner and the pull stitch is pulled one last time to tighten it and secure the graft to that corner. The same step is repeated for the anteromedial corner followed by the central pull stitch. The posteromedial FiberTapes are then retrieved from a posterolateral cannula and passed into the SwiveLock anchor to fixate the graft. The steps are then repeated for the anterolateral FiberTapes and then followed by the central FiberTapes. The graft will prevent bone to bone contact between the acromion and tuberosity.

ICMJE author disclosure forms
